# Assessment of microbiota diversity in dental unit waterline contamination

**DOI:** 10.7717/peerj.12723

**Published:** 2022-01-06

**Authors:** Yun Dang, Qian Zhang, Jing Wang, Qian Wang, Meng Han, Yuting Niu, Hua Li, Xiue Li

**Affiliations:** 1 Department of Nursing, School of Stomatology, Peking University, Beijing, China; 2 Central Laboratory, School of Stomatology, Peking University, Beijing, China; 3 Department of Preventive Dentistry, School of Stomatology, Peking University, Beijing, China; 4 The Fourth Outpatient Department, School of Stomatology, Peking University, Beijing, China; 5 Department of Pediatric Dentistry, School of Stomatology, Peking University, Beijing, China

**Keywords:** Dental unit waterlines, High-throughput sequencing, Microbiota, Water quality

## Abstract

**Background:**

Dental unit waterlines (DUWLs) provide water for handpieces, air/water syringes, and mouth-rinse water outlets. DUWL contamination can negatively affect the operating environment and public health. Therefore, it is important to elucidate the bacterial concentrations and microbial composition in the DUWLs from different dental specialties.

**Methods:**

We collected 350 5-mL dental water samples (from high-speed handpieces, air/water syringes, and mouth-rinse water outlets) from 60 dental chair units (DCUs) at a dental hospital to determine the bacterial concentrations by culture methods. Meanwhile, to investigate the diversity and community structure of microbe in the DUWLs, 17 high-quality DNA from 60 250-mL air/water syringe water samples, which were collected from the same 60 DCUs, were analyzed using 16S rDNA high-throughput sequencing.

**Results:**

The median bacterial concentration was 166 (31.5, 672.5) CFU/mL and the range was 0–3,816,000 CFU/mL. Only 42.6% of the water samples had bacterial concentrations below 100 CFU/mL. The Kruskal–Wallis *H*-test revealed that the water samples from three dental specialties had significantly different bacterial concentrations (*H* = 27.441, *P* < 0.01). High-throughput sequencing results showed significant differences in bacterial community structure between periodontics and the other two dental specialties. In the samples from three dental specialties, 508 OTUs were detected, with 160, 182 and 176 OTUs unique to the periodontics, endodontics and prosthodontics specialties, respectively. Linear discriminant analysis (LDA) effect size (LEfSe) suggested that Hydrocarboniphaga, Zoogloea, Aquabacterium, and Hydrogenophaga were enriched in the periodontics specialty; Acinetobacter, Geothrix, and Desulfovibrio were enriched in the prosthodontics specialty; and Alistipes, Clostridium XIVa, and Serratia were enriched in the endodontics specialty. Seven potentially human-pathogenic genera (Pseudomonas, Acinetobacter, Sphingomonas, Ochrobactrum, Rhizobium, Brevundimonas, and Methylobacterium) with relative abundance exceeding 1% were also detected in the DUWLs.

**Conclusions:**

The bacterial concentrations and microbial composition were influenced by different dental specialties, so a validated disinfection protocol should be used to control DUWL contamination in different dental specialties.

## Introduction

Dental unit waterlines (DUWLs) are vital components of dental chair units (DCUs) that provide water for handpieces, air/water syringes, and mouth-rinse water outlets (Hoogenkamp et al., 2020). In recent years, iatrogenic infection caused by DUWL contamination has attracted much attention (Noopan et al., 2019; Okubo et al., 2020). The first reported case of *Legionella* from DUWLs was that of an 82-year-old woman who died from Legionnaires’ disease. *L. pneumophila serogroup 1* was isolated from dental water samples and her bronchial aspirate (Ricci et al., 2012). Additionally, in 2015, nine children were infected by *Mycobacterium abscessus* after having a pulpotomy at a pediatric dentistry practice in Georgia, United States (Peralta et al., 2016). Microorganisms in the DUWLs, especially opportunistic pathogens, are potential risk factors for medically compromised or immunocompromised patients during dental care (Pankhurst, Scully & Samaranayake, 2017). Consequently, it is important to prevent DUWL contamination.

In some studies, the bacterial concentrations far exceeded the safety standards recommended by the American Centers for Disease Control and Prevention (500 colony-forming units [CFU]/mL) (Ji et al., 2016; Watanabe et al., 2016) and European Council (100 CFU/mL) (Hoogenkamp et al., 2021; Ditommaso et al., 2016). Microorganisms in the DUWLs may come from the supply water or patients’ oral cavities *via* reverse suction (Volgenant & Persoon, 2019). These microorganisms can adhere to the tube walls and colonize to form biofilms in the complex networks of thin tubes due to long periods of water stagnation (Hoogenkamp et al., 2020). Established biofilms play important roles in continued DUWL contamination by releasing fragments containing bacterial cell aggregates, and act as reservoirs of opportunistic pathogens by protecting them from antimicrobials (Dahlen, 2021). Therefore, assessing bacterial contamination and taking measures are necessary for public health.

DUWL contamination is primarily identified by bacterial culture and polymerase chain reaction (PCR) methods. However, culture may underestimate bacterial concentrations, because some bacteria exist in a viable but non-cultivable state in the environment (Hugenholtz, Goebel & Pace, 1998). Some opportunistic pathogens in the DUWLs are difficult to culture, while polymerase chain reaction methods can be used only for identifying known strains, such as *Legionella* spp., and *Pseudomonas aeruginosa* (Ditommaso et al., 2016; Ditommaso et al., 2019). All the two traditional methods are insufficient to determine the actual microbial diversity and community structure in the DUWLs (Zhang et al., 2018). Thus, risk of cross-infection by unknown bacteria in the DUWLs might be ignored. The bacterial 16S rDNA high-throughput sequencing platform can achieve the goal, with many advantages such as a fast sequencing speed, high accuracy, higher and entirety sequencing depth (Federici & Soddu, 2020). Also, some studies have applied high-throughput sequencing to elucidate microorganism diversity and community structure in freshwater system and river water (Xiong & Zhan, 2018; Zhou et al., 2021).

Therefore, this study investigated the bacterial concentrations, diversity, and community structure in the DUWLs from three dental specialties (periodontics, endodontics, prosthodontics) using culture and high-throughput sequencing technology. The aim was to clarify the risks of dental water and provide a basis for controlling contamination and establishing standards for DUWLs.

## Materials and Methods

### Water sample collection

Water samples were collected from 60 DCUs at a dental hospital: 19, 21, and 20 DCUs in the periodontics (PE-M), endodontics (EN-M), and prosthodontics (PR-M) specialties, respectively (Table S1). All of the DCUs were used for standard dental treatments, except those in the operating room. The basic information of DCUs in three dental specialties was as follows: average operating years (PE-M: 7, EN-M: 10, PR-M: 12), average patients per month (PE-M: 139, EN-M: 156, PR-M: 161) and average patients per day of DCU (PE-M: 6, EN-M: 6, PR-M: 7). The DUWLs were supplied with municipal water that meets the national standards of China, *i.e*., “Standard Examination Methods for Drinking Water—Microbiological Parameters” (GB/T 5750.12-2006) (heterotrophic plate count <100 CFU/mL at 37 °C).

*For cultivation*: Before sampling, the dental water outlets were disinfected with alcohol-soaked cotton balls, and the waterlines were flushed for 2 min before daily dental practice and 30 s after daily dental practice. 5 mL water samples were collected separately into sterilized tubes from the high-speed handpieces, air/water syringes, and mouth-rinse water outlets before and after daily dental practice (14, 21, and 20 DCUs in the periodontics, endodontics, and prosthodontics specialties, respectively). In addition, 5 mL water samples were collected only from the air/water syringes and mouth-rinse water outlets before and after daily dental practice from the other five DCUs in the periodontics specialty, because the high-speed handpieces of these DCUs were not in use.

*For high-throughput sequencing*: 250 mL water samples from air/water syringes were collected into sterilized bottles from all 60 DCUs at the same time with the 5 mL water samples collecting before daily dental practice.

### Bacterial culture

The 5 mL water samples were diluted to 1:10 and 1:100 using purified water. Then, 500 μL of each diluted solution was cultivated on a brain heart infusion (BHI) plate (BD, Franklin Lakes, NJ, USA). The plates were incubated for 48 h at 37 °C in an incubator containing 5% CO_2_. The viable bacterial counts were calculated as CFU/mL. According to China’s national standards, 100 CFU/mL was used as the threshold value.

### Statistical analysis of bacterial concentrations

Kolmogorov–Smirnov test was performed to test for normal distribution on all data. The results showed that the bacterial concentrations of three dental specialties were non-normally distributed data (*P* < 0.01). The Median (Q25, Q75) of bacterial concentrations were calculated for descriptive analysis. The Wilcoxon signed-rank test was used to compare bacterial concentrations before and after daily dental practice. The Kruskal–Wallis *H*-test was used to assess the differences in bacterial concentrations among the endodontics, periodontics, and prosthodontics specialties. *P*-values were two-sided and a *P*-value < 0.05 was considered to indicate statistical significance.

### DNA extraction

The 250 mL water samples from air/water syringes of 60 DCUs before daily dental practice were filtered through a 0.2-μm pore polycarbonate filter (Millipore, Billerica, MA, USA), which was then stored in phosphate-buffered saline at 4 °C. Microorganism precipitates were obtained by centrifugation at 12,000 rpm for 10 min; the filter membrane was discarded. Bacterial genomic DNA was extracted using a Takara MiniBEST Bacterial Genomic DNA Extraction Kit (Takara, Dalian, China), following the manufacturer’s protocol. High-quality DNA with OD_260/280_ = 1.8–2.0, concentration >5 ng/μL, and no degradation with agarose gel electrophoresis (from 17 air/water syringe water samples) was stored at −80 °C until being used for molecular applications.

### High-throughput sequencing

The high-quality extracted DNA was used for further PCR amplification with primers targeting the V3–V4 hypervariable regions of bacterial 16S rRNA genes. The primers were 314F (5-CCTACGGGRSGCAGCAG-3) and 806R (5-GGACTACVVGGGTATCTAATC-3) with an 8-bp unique barcode. PCR amplification was performed using a KAPA HiFi HotStart ReadyMix PCR kit (Roche, Basel, Switzerland) using a 20 μL reaction mixture. Thermal cycling conditions were as follows: an initial denaturation at 95 °C for 3 min, 27 cycles of 95 °C for 30 s, 55 °C for 30 s, and 72 °C for 45 s, and a final extension at 72 °C for 10 min. The PCR products were examined using 2% agarose gel electrophoresis and purified using an AxyPrep Gel Extraction kit (Axygen, Union City, CA, USA). Equimolar samples were pooled together for sequencing using the Illumina MiSeq PE250 platform (Shanghai Realgene Biotech, Shanghai, China). The raw sequence data had been submitted to NCBI under accession number PRJNA690183.

### Statistical analysis of sequencing data

The paired-end reads were merged based on the overlap base pairs using PANDAseq software (Masella et al., 2012). Clean merged reads were obtained with an average quality >20 and base *N* < 3. The sequences were clustered into operational taxonomic units (OTUs) at a 97% similarity level using USEARCH after removing chimeras and singletons (Edgar, 2013). Based on optimized OTUs, Alpha-diversity indices (including Chao1, Observed species, Shannon, and Simpson indices) were calculated by extracting the same reads from each sample and compared using the Kruskal–Wallis *H*-test to determine the significance of differences in bacterial diversity among the dental specialties. The taxonomic annotations (phylum, class, order, family, and genus) of OTUs were identified using the RDP database. Principal coordinates analysis (PCoA) and hierarchical cluster analysis were performed with the nonparametric analysis of variance using distance matrices (ADONIS) to examine the bacterial communities in three dental specialties.

## Results

### Microbial culture of water samples

A total of 350 dental water samples were collected. The median bacterial concentration was 166 (31.5, 672.5) CFU/mL and the range was 0–3,816,000 CFU/mL. Only 42.6% of the water samples had bacterial concentrations below the threshold of China’s national drinking-water standards (heterotrophic plate counts <100 CFU/mL).

Differences in bacterial concentrations among the samples from the periodontics, endodontics, and prosthodontics specialties were significant (Table 1). There was no significant difference in bacterial concentrations between samples before and after daily dental practice (Table 2).

**Table 1:** Comparison of bacterial concentrations among the three specialties.

Specialty	Sample numbers	Median (Q25, Q75)	*H*	*P*
Periodontics	104	335 (116.5, 1,060)	27.441	<0.01
Endodontics	126	79 (20, 410)		
Prosthodontics	120	141 (20, 540)		

**Table 2:** Comparison of bacterial concentrations between samples before and after daily dental practice.

Sampling time	Sample numbers	Median (Q25, Q75)	*Z*	*P*
Before daily dental practice	175	192 (30, 880)	−1.626	0.104
After daily dental practice	175	160 (32, 630)		

### Overview of sequencing results

After quality control and filtering, 603,296 clean reads were obtained from 17 dental water samples (6, 5, and 6 samples from the periodontics, endodontics, and prosthodontics specialties, respectively) with an average of 35,488 ± 1,173 sequences per sample (range: 33,120–37,988), representing 7,130 OTUs. On average, 419 OTUs were detected in each sample (range: 262–562).

### Bacteria composition in the periodontics, endodontics, and prosthodontics specialties

**Figure 1:**
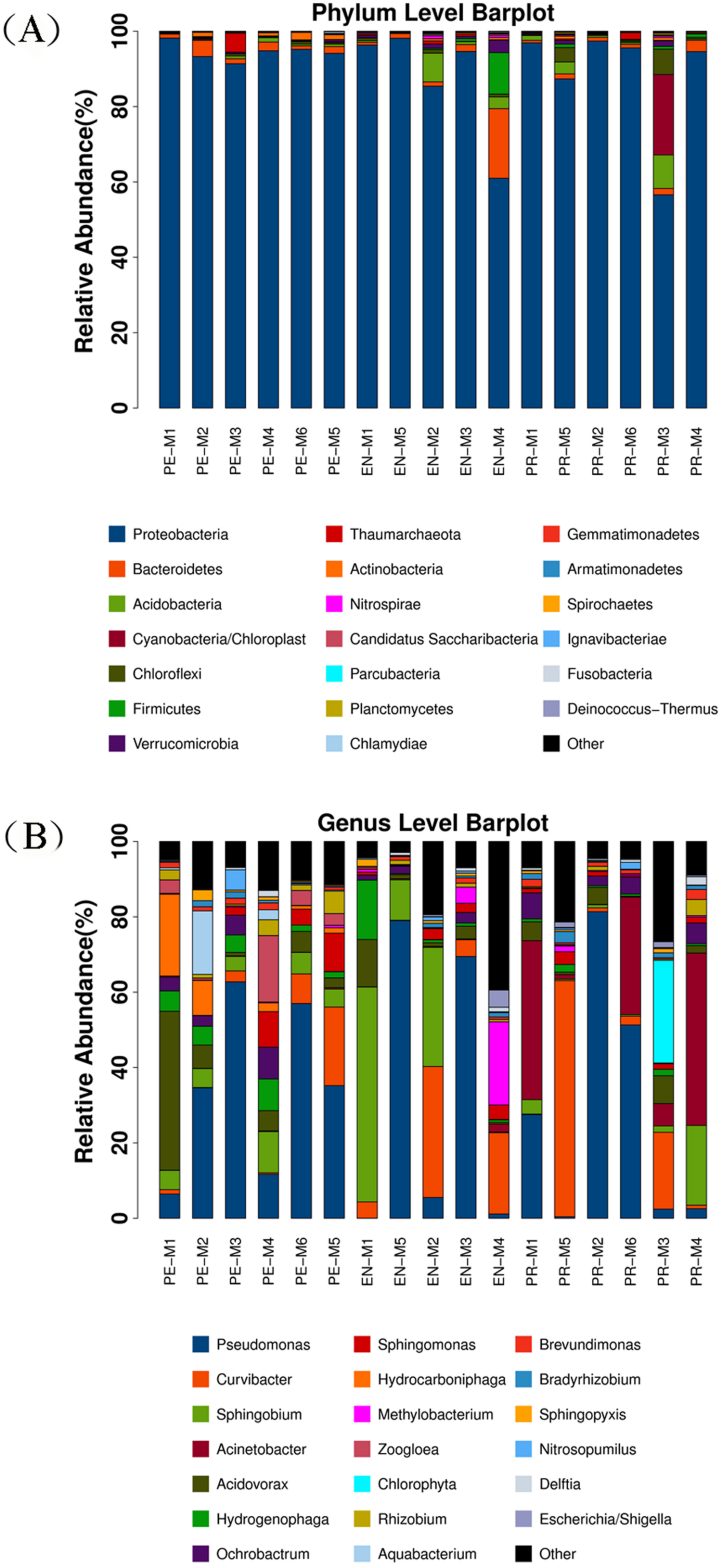
Dominant bacterial communities in 17 water samples from the periodontics (PE-M), endodontics (EN-M), and prosthodontics (PR-M) specialties. (A) Dominant bacterial communities at the phylum level in different specialties. (B) Dominant bacterial communities at the genus level in different specialties.

The main phylum, with a relative abundance more than 85%, was Proteobacteria (Fig. 1A). Other phyla included Bacteroidetes, Acidobacteria, Chloroflexi, and Firmicutes. Twenty genera were detected in the 17 water samples from the periodontics (PE-M), endodontics (EN-M), and prosthodontics (PR-M) specialties. Seven potentially human-pathogenic genera with relative abundances exceeding 1% were detected in the 17 samples from three dental specialties, including Pseudomonas (relative abundance: 31.08%), Acinetobacter (7.64%; 21.05% for PR-M, <1% for PE-M and EN-M), Sphingomonas (2.68%), Ochrobactrum (2.71%), Rhizobium (1.47%; 2.61% for PE-M, 1.04% for PR-M, <1% for EN-M), Brevundimonas (1.05%; 1.13% for PE-M, 1.26% for PR-M, <1% for EN-M), and Methylobacterium (1.88%; 5.48% for EN-M, <1% for PE-M and PR-M) (Fig. 1B). Other genera included Sphingobium (9.61%), Curvibacter (10.93%), Acidovorax (5.95%), Hydrogenophaga (3.00%), Hydrocarboniphaga (2.10%), Zoogloea (1.71%), and Aquabacterium (1.22%). Hydrocarboniphaga was detected only in the periodontics specialty, while Zoogloea and Aquabacterium were detected in the periodontics and prosthodontics specialties.

### Bacterial diversity in the periodontics, endodontics, and prosthodontics specialties

**Figure 2:**
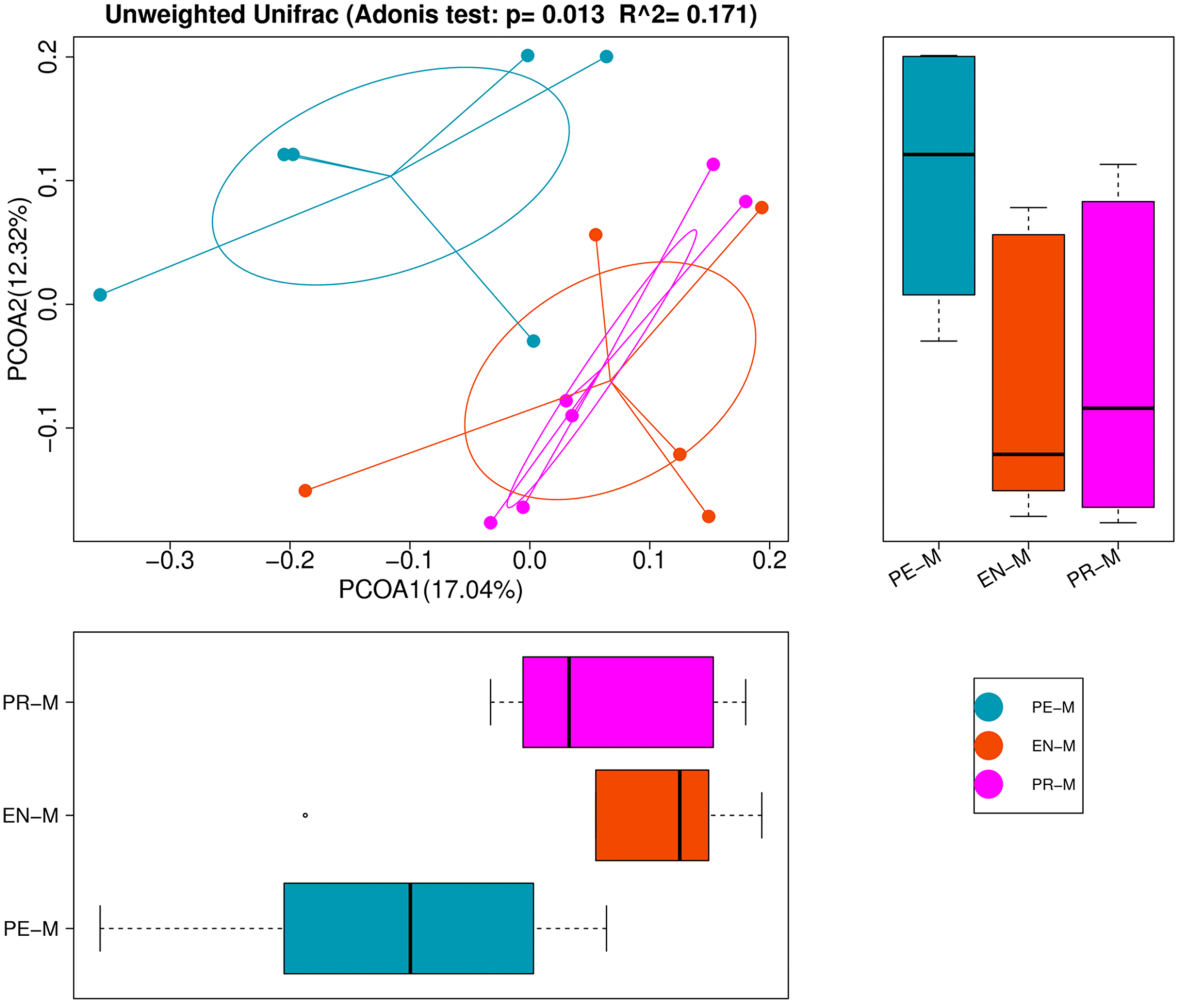
Principal coordinates analysis (PCoA) and analysis of variance using distance matrices (ADONIS) of the 17 water samples from the periodontics (PE-M), endodontics (EN-M), and prosthodontics (PR-M) specialties.

The alpha-diversity indices, which estimated bacteria richness (Chao1 and observed species indices) and diversity (Shannon and Simpson indices), are presented in Table 3. There were no significant differences in diversity indices among the periodontics, endodontics, and prosthodontics specialties (*P* > 0.05). However, PCoA and ADONIS showed significant differences in bacterial community structure among the 17 samples from three dental specialties (Fig. 2). Samples from the endodontics and prosthodontics specialty clustered together, whereas samples from the periodontics specialty were far removed from the other two, indicating differing community structures among the different dental specialties.

**Table 3:** Comparison of Alpha-diversity indices among the three specialties.

Alpha-diversity	Periodontics	Endodontics	Prosthodontics	*P*
Chao1	446.49 ± 106.82	470.13 ± 123.29	459.14 ± 89.05	0.953
Observed species	343.50 ± 90.54	383.20 ± 121.75	371.33 ± 98.26	0.731
Shannon	4.41 ± 0.69	3.77 ± 1.75	3.99 ± 1.10	0.594
Simpson	0.88 ± 0.61	0.69 ± 0.22	0.79 ± 0.81	0.159

### Different bacterial communities in the periodontics, endodontics, and prosthodontics specialties

**Figure 3:**
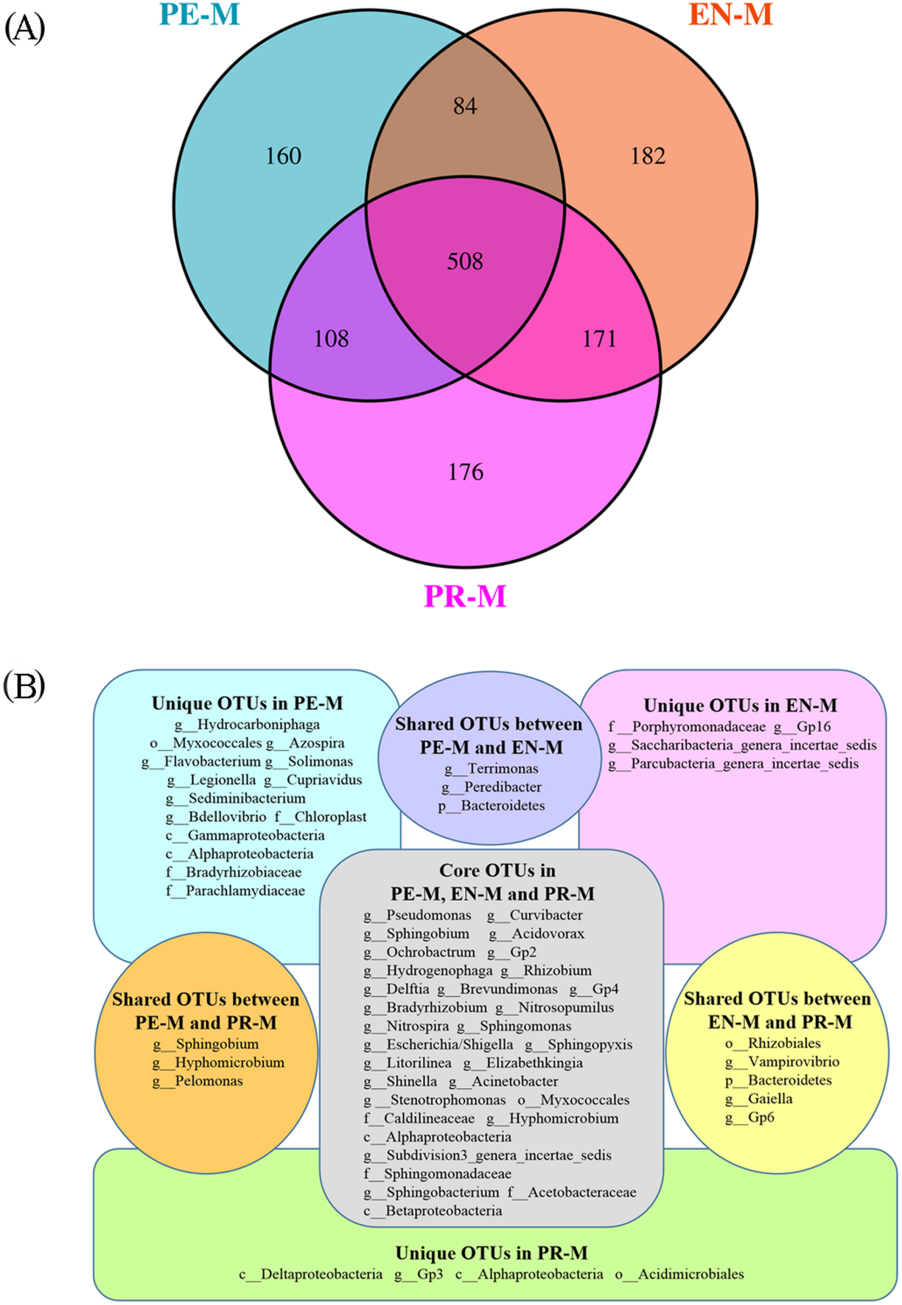
The shared and unique OTUs among the periodontics (PE-M), endodontics (EN-M), and prosthodontics (PR-M) specialties. (A) Venn diagram of the numbers of shared and unique OTUs among the three specialties. (B) The taxonomic nomenclature of shared and unique OTUs among the three specialties. The OTUs in (B) were present in at least 50% of the samples and had a mean relative abundance of >0.01% of all sequences.

There were 860, 945, and 963 bacterial OTUs in the periodontics, endodontics, and prosthodontics specialties, respectively, and 508 OTUs were detected in all samples of the three specialties (Fig. 3A). The OTUs with detection frequencies exceeding 50% were showed in Fig. 3B. There were 160, 182, and 176 OTUs unique to the periodontics, endodontics, and prosthodontics specialties, respectively. OTUs unique to the periodontics specialty included Sediminibacterium, Solimonas, Legionella, Bdellovibrio, Hydrocarboniphaga, Flavobacterium, Cupriavidus, and Azospira; those unique to the endodontics specialty included Gp16, Parcubacteria genera incertae sedis, and Saccharibacteria genera incertae sedis; only Gp3 was unique to the prosthodontics specialty. The periodontics specialty shared 592 and 716 OTUs with the endodontics and prosthodontics specialties, respectively, while the endodontics and prosthodontics specialties shared 679 OTUs (Fig. 3A).

**Figure 4:**
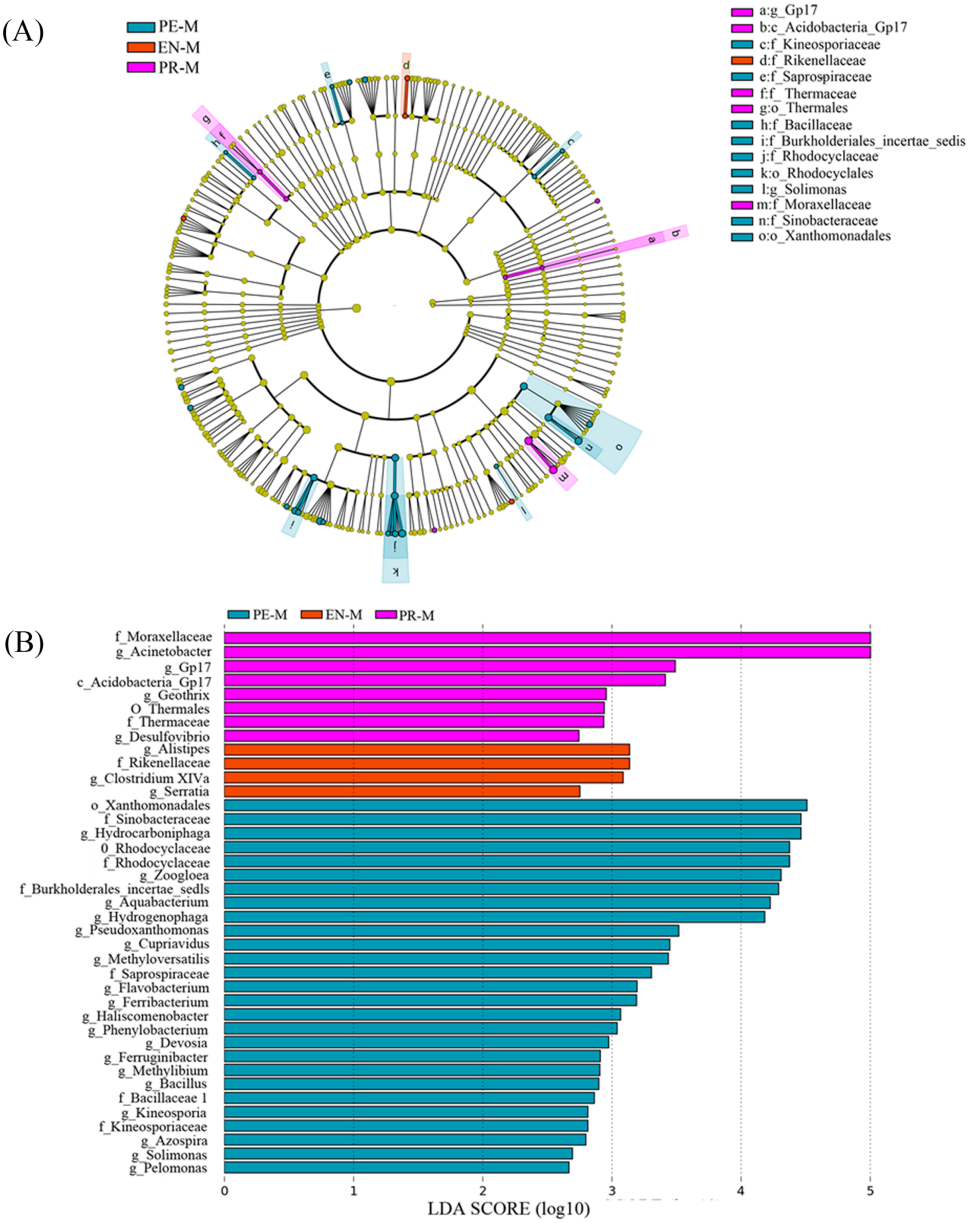
The distribution of microbial organisms in the periodontics (PE-M), endodontics (EN-M), and prosthodontics (PR-M) specialties. (A) Cladogram analysis. (B) Linear discriminant analysis (LDA) effect size (LEfSe).

Linear discriminant analysis (LDA) effect size (LEfSe) was used to identify the presence and effect size of region-specific OTUs among the water samples of three specialties (Fig. 4). The results suggested that Acinetobacter, Gp17, Geothrix, and Desulfovibrio were enriched in the prosthodontics specialty; Alistipes, Clostridium XIVa, and Serratia were enriched in the endodontics specialty; and Hydrocarboniphaga, Zoogloea, Aquabacterium, and Hydrogenophaga were enriched in the periodontics specialty.

## Discussion

DUWL Contamination puts patients at potential risk during dental care. Therefore, this study analyzed the bacterial concentrations, diversity, and community structure in DUWLs of periodontics, endodontics, and prosthodontics specialties to provide a basis for controlling contamination and establishing standards for DUWLs.

Bacteria can be easily colonized in the DUWLs environment. Previous studies had showed that the bacterial concentrations can reached 1.4 × 10^5^ and 1.8 × 10^6^ CFU/mL (Watanabe et al., 2016; Ji et al., 2018). Also in our study, bacterial concentrations ranged from 0 to 3,816,000 CFU/mL, and only 42.6% of water samples met China’s national drinking-water standards. And the bacterial concentrations of water samples differed significantly in three specialties. We also compared water samples before and after daily dental practice and found no significant difference in bacterial concentrations. The probable cause of the result was that although flushing during dental care could reduce bacterial concentrations to an extent (Watanabe et al., 2008), suck-back of saliva and blood still made contamination a serious concern (Spagnolo, Sartini & Cristina, 2020). Some main factors of DUWL contamination were the small lumen size (0.5–2 mm) and materials of tubes, high surface-to-volume ratio (6:1), slow water flow during working hours and water stagnation outside of working hours (Spagnolo, Sartini & Cristina, 2020). These factors allowed bacteria to be easily colonized in the DUWLs environment and to contaminate output water. If dental water was not appropriately treated, patients’ health could be at risk.

In our study, Proteobacteria was a prevalent phylum in DUWLs with the relative abundance over 85% of the total phyla, which was similar with previous studies which showed that Proteobacteria was also the most abundant phylum in biofilm samples (Fan et al., 2021) and in dental water (Costa et al., 2015; Yoon & Lee, 2019). Meanwhile, because of the high tolerance to chlorine compared with other phylum, Proteobacteria was also the most commonly detected phylum in water distribution systems (Huang et al., 2014; Holinger et al., 2014). The predominant bacterial genera with relative abundance more than 5% detected in our study were Pseudomonas, Curvibacter, Sphingobium, Acinetobacter and Acidovorax. Among these five genera, Pseudomonas and Acinetobacter were human-pathogenic bacteria, which were also detected in other researches (Costa et al., 2015; Zhang et al., 2018; Yoon & Lee, 2019; Fan et al., 2021). Pseudomonas could colonize and form biofilm in plastic waterlines (Spagnolo, Sartini & Cristina, 2020). *Pseudomonas* spp., especially *Pseudomonas aeruginosa*, could grow in low-nutrient environment and often exhibit resistance to antimicrobial agents and disinfectants (Rodrigues et al., 2017). Acinetobacter was also a well-known cause of nosocomial infections, and exhibited a high degree of drug resistance (Harding, Hennon & Feldman, 2018). These pathogens may put medically compromised or immunocompromised individuals at risk of cross-infection during dental care. Besides, endotoxins released from gram-negative pathogens in the DUWLs might introduce allergic airway reaction (Coleman et al., 2009) and increase the release of proinflammatory cytokines in gingival tissue during oral surgery (Putnins, Di Giovanni & Bhullar, 2001). Therefore, a disinfection protocol should be used to reduce DUWL contamination. Chemical disinfectants, such as peracetic acid, hydrogen peroxide, sodium hypochlorite, chlorine dioxide, chlorhexidine, 2,100 ppm ozone, iodine cartridges, and super-oxidized water, can be used either periodically or continuously to treat DUWL biofilms (Spagnolo, Sartini & Cristina, 2020; Cicciu, 2020). Physical measures, including filtration, flushing, and reverse osmosis, should also be combined with disinfection measures. In addition, the scheduled technical maintenance of dental units and monitoring the quality of dental water are equally important. The pathogenic mechanisms, independent factors, and drug resistance of pathogenic bacteria should also be explored further to provide a basis for DUWL disinfection.

Dental practices and treated patients might be important factors for bacterial contamination in different dental specialties. Patients with different oral diseases are treated in different dental specialties with different dental practices. The oral microbiome is the driving factor of oral diseases. For example, *Porphyromonas gingivalis*, which is the main pathogen responsible for periodontitis, and *Streptococcus mutans*, which gives rise to dental caries (He et al., 2015), may enter DUWLs in the periodontics and endodontics specialties *via* suck-back. As stated before, the bacterial concentrations of water samples differed significantly in three specialties of our study. The periodontics specialty had a significantly different DUWL bacterial community structure from the endodontics and prosthodontics specialties and harbored more genera that were not found in other two specialties. Additionally, the relative abundance of four pathogenic genera (Acinetobacter, Rhizobium, Brevundimonas and Methylobacterium) also differed among the three dental specialties. The results were consistent with Zhang et al. (2018), which reported that the water quality in an endodontics specialty was better than that in a periodontics specialty. Yoon & Lee (2019) also found similar bacterial diversities in the dental water of pedodontics and periodontics specialties, while the dental water of a private dental clinic had distinct bacterial diversity. It prompted that bacteria may have different distributions in water samples among the dental specialties, possibly because of the suck-back of saliva and blood during dental care. Although oral microorganisms have lower survival in the DUWLs than oral cavity, the colonization and distribution of other water microorganisms in the DUWLs may still be affected by short-term interactions with oral microorganisms. In addition, differences in hydrodynamic stress among the three specialties may also affect the variation in microbial communities and bacterial concentrations by impacting the composition, density, and structure of biofilms (Hoogenkamp et al., 2020). Therefore, a validated disinfection protocol should be used to control DUWL contamination in different specialties. In addition, anti-retraction valves are usually fitted distally to handpieces and air/water syringes to prevent suck-back. However, Ji et al. (2016) pointed that 51.72% of DCUs failed the retraction evaluation because of anti-retraction valve failure after a few months’ use. Dental staff should monitor the efficacy of anti-retraction valve through a retraction measurement device and the volume of water retraction should not exceed 40 μL (Ji et al., 2016).

## Conclusions

Our study used culture and high-throughput sequencing to analyze the bacterial concentrations, diversity, and community structure in the DUWLs. The bacterial concentrations ranging from 0 to 3,816,000 CFU/mL revealed DUWL contamination required more attention. Proteobacteria was the most dominant phylum in the DUWLs. Among the predominant bacterial genera detected in our study, Pseudomonas and Acinetobacter were human-pathogenic bacteria. Therefore, disinfection measures are required to control DUWL contamination. In addition, the scheduled technical maintenance of dental units and monitoring the quality of dental water are equally important. The bacterial concentrations of dental water samples differed significantly in three specialties and the periodontics specialty had a significantly different DUWL bacterial community structure from the endodontics and prosthodontics specialties, possibly because of the short-term interactions with oral microorganisms due to suck-back and differences in hydrodynamic stress among the three specialties. The results suggested that a validated disinfection protocol should be used to control DUWL contamination in different specialties.

## Supplemental Information

10.7717/peerj.12723/supp-1 Supplemental Information 1 Samples information in the study.

10.7717/peerj.12723/supp-2 Supplemental Information 2 The raw data for Table 1 and Table 2.

10.7717/peerj.12723/supp-3 Supplemental Information 3 The overall relative abundance (%) top bacteria at phylum, class, order, family and genus levels and that among three groups of DUWL dental water samles (reltive abundance >0.1%).

## Additional Information and Declarations

### Data Availability

The following information was supplied regarding data availability: Raw data are provided as a Supplemental File. The raw sequences are available at NCBI (PRJNA690183) and figshare: Dang, Yun (2021): rawdata.rar. figshare. Dataset. DOI 10.6084/m9.figshare.16543935.v1.
