# Supplementary material for: Assessment of microbiota diversity in dental unit waterline contamination
Source: PeerJ. 2022 Jan 6;10:e12723. doi: 10.7717/peerj.12723 (PMC8743008; doi:10.7717/peerj.12723)
Supplement: Supplemental Information 1 [file peerj-10-12723-s001.docx]

**Supplemental Table S1 : Samples information in the study.**

| **Department** | **DCU numbers** | **Sampling sites** | | | **Sampling time** | **Sample numbers** |
| --- | --- | --- | --- | --- | --- | --- |
|  |  | High-speed handpiece | Air/water syringe | Mouth-rinse water |  |  |
| Periodontics | 5 | － | √ | √ | Morning and Afternoon | 5*2*2=20 |
|  | 14 | √ | √ | √ |  | 14*3*2=84 |
| Endodontics | 21 | √ | √ | √ |  | 21*3*2=126 |
| Prosthodontics | 20 | √ | √ | √ |  | 20*3*2=120 |
| Total | 60 |  | | |  | 350 |
